# CLINICOPATHOLOGIC CORRELATION OF GEOGRAPHIC ATROPHY SECONDARY TO AGE-RELATED MACULAR DEGENERATION

**DOI:** 10.1097/IAE.0000000000002461

**Published:** 2019-02-06

**Authors:** Miaoling Li, Rosa Dolz-Marco, Carrie Huisingh, Jeffrey D. Messinger, Richard M. Feist, Daniela Ferrara, K. Bailey Freund, Christine A. Curcio

**Affiliations:** * Department of Ophthalmology and Visual Sciences, School of Medicine, University of Alabama at Birmingham, Birmingham, Alabama;; † State Key Laboratory of Ophthalmology, Zhongshan Ophthalmic Center, Sun Yat-sen University, Guangzhou, China;; ‡ Vitreous Retina Macula Consultants of New York, New York, New York;; § LuEsther T Mertz Retinal Research Center, Manhattan Eye, Ear and Throat Hospital, New York, New York;; ¶ Unit of Macula, Oftalvist Clinic, Valencia, Spain;; ** Retina Consultants of Alabama, Birmingham, Alabama;; †† Genentech, South San Francisco, California; and; ‡‡ Department of Ophthalmology, New York University School of Medicine, New York, New York.

**Keywords:** age-related macular degeneration, basal laminar deposits, basal linear deposits, drusen, subretinal drusenoid deposits, external limiting membrane, outer retina, photoreceptors, retinal pigment epithelium, Müller cells, optical coherence tomography, fundus autofluorescence, histology, complete retinal pigment epithelium and outer retinal atrophy, geographic atrophy

## Abstract

**Purpose:**

In an eye with geographic atrophy (GA) secondary to age-related macular degeneration, we correlated ex vivo histologic features with findings recorded in vivo using optical coherence tomography (OCT), near-infrared reflectance imaging, and fundus autofluorescence.

**Methods:**

In the left eye of an 86-year-old white woman, in vivo near-infrared reflectance and eye-tracked OCT B-scans at each of 6 clinic visits and a baseline fundus autofluorescence image were correlated with high-resolution histologic images of the preserved donor eye.

**Results:**

Clinical imaging showed a small parafoveal multilobular area of GA, subfoveal soft drusen, refractile drusen, hyperreflective lines near the Bruch membrane, subretinal drusenoid deposit (reticular pseudodrusen), and absence of hyperautofluorescent foci at the GA margin. By histology, soft drusen end-stages included avascular fibrosis with highly reflective cholesterol crystals. These accounted for hyperreflective lines near the Bruch membrane in OCT and plaques in near-infrared reflectance imaging. Subretinal drusenoid deposit was thick, continuous, extracellular, extensive outside the fovea, and associated with distinctive retinal pigment epithelium dysmorphia and photoreceptor degeneration. A hyporeflective wedge corresponded to ordered Henle fibers without cellular infiltration. The external limiting membrane descent, which delimits GA, was best visualized in high-quality OCT B-scans. Retinal pigment epithelium and photoreceptor changes at the external limiting membrane descent were consistent with our recent histologic survey of donor eyes.

**Conclusion:**

This case informs on the extent, topography, and lifecycle of extracellular deposits. High-quality OCT scans are required to reveal all tissue features relevant to age-related macular degeneration progression to GA, especially the external limiting membrane descent. Histologically validated signatures of structural OCT B-scans can serve as references for other imaging modalities.

## Abstract 2

Supplemental Digital Content is Available in the Text.

Direct clinicopathologic correlation of an eye with geographic atrophy secondary to age-related macular degeneration provides histologic correlates of features commonly seen by optical coherence tomography, such as end-stages of drusen, subretinal drusenoid deposit, plaques near the Bruch membrane, and hyporeflective wedge.

Age-related macular degeneration (AMD) is the main cause of central vision loss in the elderly of industrialized countries. Treatment exists for neovascular AMD, but not for the atrophic end-stage, geographic atrophy (GA). Geographic atrophy is variably defined, depending on the imaging modality used. Based on optical coherence tomography (OCT), a new term proposed for GA is complete retinal pigment epithelium (RPE) and outer retinal atrophy (cRORA).^1^ Recent clinical trials for agents targeting enlargement of GA^2–4^ provided standardized longitudinal imaging of well-defined patient groups useful for elucidating an accurate natural history of GA and with it, insight into underlying disease mechanisms.

Remarkably, subcellular-level detail is available in commercially available OCT, thanks to signal averaging and point-of-capture quality control, eye-tracking, and integration with multiple imaging techniques.^5^ Based on histology,^6^ we proposed as the border of atrophy in the photoreceptor layer a curve of the external limiting membrane (ELM) toward the Bruch membrane (BrM, ELM descent).^7,8^ A progressive RPE dysmorphia toward this border thickened the reflective RPE band and was suggested as an OCT biomarker^7,8^ and a correlate to hyperautofluorescence at the GA margin.^8,9^ With longitudinal OCT, we showed that during GA enlargement, Müller cells scrolled up the outer retina at the ELM descent, in advance of RPE degeneration.^10,11^

Recent high-resolution histology of newly described and better-defined extracellular lesions, deployed in precise layers (Figure 1), has informed clinical image interpretation. Subretinal drusenoid deposit (SDD), also called reticular pseudodrusen, is located between the RPE and photoreceptors^12^ and associates with progression from intermediate AMD to GA and Type 3 neovascularization (retinal angiomatous proliferation).^13–16^ Basal laminar deposit (BLamD), a thickened basement membrane material between the RPE and its native basal lamina (BL), is considered diagnostic for AMD when continuous under the fovea.^17–20^ Soft drusen and basal linear deposit (BLinD) are lump-and-layer forms of the same AMD-specific lipid-rich material, located between the RPE-BL and the inner collagenous layer of BrM.^21,22^ Deposits are dynamic, showing phases of growth, regression, and persistence visible by longitudinal in vivo eye-tracked OCT.^18,20,23–26^ Furthermore, the topographies of SDD and BLinD follow those of rods and cones, respectively, suggesting dysregulation of previously unknown pathways specific to each photoreceptor type.^13,27^

**Fig. 1.**
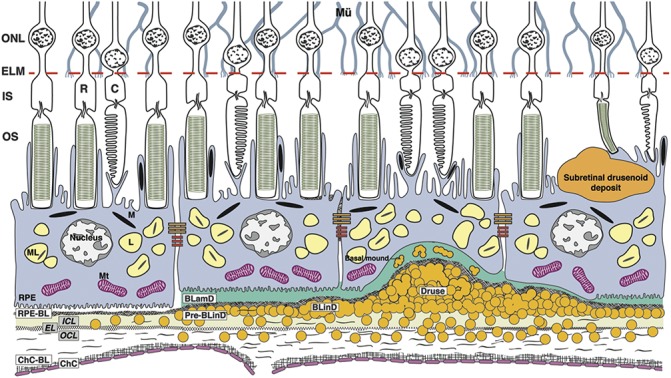
Age-related macular degeneration, by the layers. Mü, Müller glia; ONL, outer nuclear layer; R, rods; C, cones; ELM, external limiting membrane; IS, inner segments of photoreceptors; OS, outer segments of photoreceptors; RPE, retinal pigment epithelium; M, melanosome; ML, melanolipofuscin; Mt, mitochondria; RPE-BL, RPE basal lamina; BLamD, basal laminar deposit; BLinD, basal linear deposit; ICL, inner collagenous layer; EL, elastic layer; OCL, outer collagenous layer; ChC-BL, ChC basal lamina; yellow circles, lipoprotein particles. The Bruch membrane (BrM) consists of the ICL, EL, and OCL. Soft drusen and BLinD are two forms (lump and layer) of the same AMD-specific extracellular deposit. Basal mound is soft druse material within BLamD. Basal laminar deposit is thickened RPE-BL. Subretinal drusenoid deposit localizes to the subretinal space (between photoreceptors and RPE).

Clinicopathologic correlation, i.e., histology of eyes imaged during life, can both validate diagnostic technology with anatomical ground truth and probe disease pathogenesis. In this study, we describe the first clinicopathologic correlation of an eye with GA secondary to AMD for which serial OCT and near-infrared reflectance (NIR) are available, along with baseline fundus autofluorescence (FAF). The index eye can be compared with the fellow eye that progressed to Type 3 neovascularization,^28^ our recent histologic surveys of GA in donor eyes,^7,8^ and a clinicopathologic correlation of macular atrophy due to mixed causes in AMD.^29^ Our current results demonstrate the subcellular detail available for clinical decision-making today through OCT-anchored imaging.

## Methods

### Compliance

Studies were approved by the institutional review board at the University of Alabama at Birmingham and adhered to the Tenets of the Declaration of Helsinki.

### Terminology for Optical Coherence Tomography and Histologic Layers and Regions

The index eye met proposed criteria^1^ for cRORA, i.e., atrophy ≥250-*µ*m diameter involving outer retinal layers and RPE. We use consensus OCT terminology^30^ with the addition of the RPE-BL–BrM (RBB) band.^31^ The presence of choroidal hypertransmission defined the presence of cRORA/GA on structural cross-sectional OCT scans. To delineate the areas of GA, these areas were then mapped on the NIR image. As recommended,^32,33^ AMD pathology is most easily described if BrM is considered as a subendothelial space with three layers (inner collagenous, elastic, and outer collagenous), and the RPE-BL or BLamD is considered with the RPE. Between the RPE-BL (or BLamD) and the inner collagenous layer is the sub–RPE-BL space. We refer to the combination of SDD, RPE, BLamD, drusen/BLinD, and BrM as the RPE-deposit complex.^34^ For macular regions, we refer to the (rod-free) fovea, parafovea, and perifovea (radii of outer boundaries 250, 1,250, and 2,750 *µ*m, respectively).^35^

### Clinical Course

An 86-year-old white woman with GA in her left eye and Type 3 neovascularization in her right eye, both secondary to AMD, was referred to a private retina practice for evaluation and treatment. She had undergone chemotherapy, radiotherapy, and upper lobectomy for lung cancer in 2002 but was not a smoker. Her ocular history included open-angle glaucoma stable under acetazolamide, dorzolamide, and timolol therapy with 20/20 visual acuity in both eyes. In the left eye, pigment changes were noted 3 years prior and soft drusen 2 years before referral to a retina specialist, which was prompted by suddenly decreased visual acuity in her right eye (20/70) and progressive vision loss in her left eye (20/200). Because of a diagnosis of Type 3 neovascular AMD in the right eye,^28^ she initiated anti–vascular endothelial growth factor therapy. At this time, the left eye was found to have GA without neovascularization, and it was not treated. From November 2012 to July 2013, she received in the right eye a total of 6 intravitreal injections of bevacizumab (1.25 mg/0.05 mL) with optimal response and resolution of the exudation. Available for review were an FAF baseline image from the left eye, eye-tracked OCT B-scans, and NIR for both eyes at each of the six examinations. The last clinical evaluation, on September 2013, lacked OCT imaging.

Four months after the July 2013 visit with OCT, the patient died of a cerebrovascular accident. Both eyes were recovered for research 7.5 hours after death by personnel of the Alabama Eye Bank.^28^ The clinical diagnosis of GA was supported histologically by a 1,127-*µ*m-wide absence of continuous RPE, in the presence of BLinD and drusen, and the absence of neovascularization.

### Clinical Analysis of Atrophy Borders and Layer Thicknesses

A quantitative analysis using in vivo eye-tracked OCT scans was performed at four anatomical points (at 100 and 500 *µ*m at the nonatrophic and atrophic side of the ELM descent) as described.^29^ Measurements were taken in consecutive visits (Visit 1: November 2012: 12 months before death; and Visit 2: July 2013, 4 months before death). For comparison with histology, thicknesses were measured for outer retina (inner boundary of the outer plexiform layer [OPL] to BrM), OPL to ELM, outer nuclear layer (ONL), RBB, and subfoveal choroid (from the outer RBB boundary to the inner surface of the scleral–choroidal boundary) with the manual caliper in the OCT device software (Spectralis Reviewer, version 6.9.5.0). The RBB thickness was also measured in the perifovea at 2,000 *µ*m nasal and temporal to the fovea (17 different locations per side). Visibility of the ELM descent was assessed in all available OCT volumes (6 visits, 10 B-scans per visit). We previously showed^29^ that the number of images averaged for each B-scan in the automatic real time (ART) function and the quality value in decibels (dB) impact visibility of small features, and thus, these values were recorded.

### Ex Vivo Imaging and Histopathologic Correlation With In Vivo Optical Coherence Tomography

The preserved globe underwent ex vivo OCT imaging before histologic processing, as described^36^ (See **Figure 1, Supplemental Digital Content 1**, http://links.lww.com/IAE/A954, compares in vivo and ex vivo OCT). Although our previous studies used eye-tracking to align in vivo and ex vivo OCT scans, for this eye, such tracking was not possible, and we correlated histology to in vivo scans as follows. An 8-mm-diameter full-thickness tissue punch centered on the fovea was post-fixed with osmium tannic acid paraphenylenediamine to preserve extracellular lipids^37^ and embedded in epoxy resin. The tissue punch was oriented in the embedding mold assisted by a drawing to ensure sectioning in a strictly superior-to-inferior direction. The polymerized epoxy block was reviewed under a dissecting microscope. A reference line drawn across the block face between the optic nerve and fovea was used to align the block parallel to the plane of sectioning in an ultramicrotome (UltraCut, Leica Microsystems, Buffalo Grove, IL). The ex vivo OCT volume was reviewed to identify retinal contours, pathologies of interest, and superior-to-inferior sequence of occurrence within the volume. Cumulative distances were calculated using this OCT volume and a digital counter on the ultramicrotome. Three-micrometer sections were removed and reviewed with a 10× microscope objective to seek features expected from the OCT volume and initiate the collection of serial 0.8-*µ*m-thickness sections that were then stained with toluidine blue. A total of 45 sections were obtained (mean distances between sections: 88 total, 20–280 *µ*m apart). The location of each histologic section was aligned to the ex vivo OCT, thus allowing for point-by-point in vivo/ex vivo correlation.

To provide seamless zoom-in for details and zoom-out for context without switching between objectives, sections were scanned in their entirety using a 60× oil-immersion objective (numerical aperture = 1.4), a robotic microscope stage, and slide scanning software (Olympus VSI 120, CellSens; Olympus, Center Valley, PA). Scanned images were scaled for tissue units and centered,^8,38,39^ then viewed on a monitor at magnifications up to 1,240× using ImageJ software (https://imagej.nih.gov/ij/download.html). Photomicrographs were composited for figures with adjustments for exposure, contrast, and background color correction (Photoshop CS6; Adobe Systems San Jose, CA).

### Histopathologic Analysis of Cellular Phenotypes and Layer Thicknesses

**Fig. 2.**
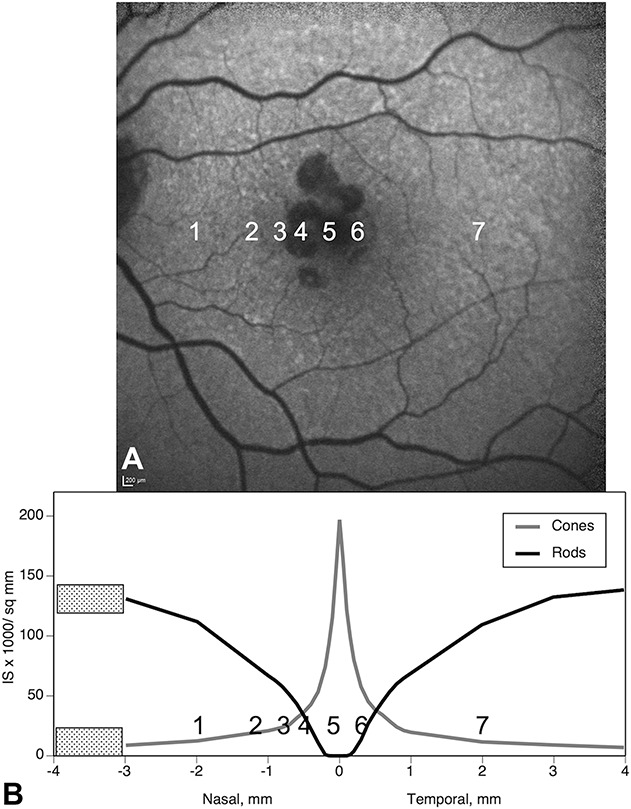
Guide to assessment locations in Table 2. In Table 2, layer thicknesses are displayed for 7 locations from nasal to temporal. Locations 1 and 7 compare SDD with different clinical appearances. Locations 2 to 6 compare changes across the border of atrophy as defined by the descent of the ELM. **A.** Baseline autofluorescence image of the index case. **1.** Area of SDD (reticular pseudodrusen), nasal perifovea; (**2** and **3**) 500 and 100 *µ*m, respectively, from the ELM descent in nonatrophic retina, nasal parafovea; (**4**) 100 *µ*m from the ELM descent, in the GA area, both sides pooled, nasal parafovea; (**5** and **6**) 500 and 100 *µ*m, respectively, from the ELM descent, nonatrophic retina, fovea; and (**7**) area of “target-appearance” SDD, temporal perifovea. **B.** Distribution of cone and rod photoreceptor inner segments (IS) per mm^2^ in adult human macula,^62^ as determined from retinal flatmounts. Layer thickness assessment locations in the index eye are indicated. Stippling indicates the optic nerve head.

As described,^7,8,39^ we determined the frequency of cellular and laminar phenotypes and measured layer thicknesses in seven regions (Figure 2): two perifoveal regions with distinct NIR presentation and five regions at defined distances from the borders of GA, keeping foveal locations separate. The borders' analysis entailed assessments on the nonatrophic and atrophic sides of ELM descents, as described above for OCT,^7,8,39^ in 7 sections with 16 ELM descents. At the seven specified points, we assessed phenotypes and measured thicknesses of SDD, RPE, BLamD, sub–RPE-BL space, BrM (as defined above), choriocapillaris (ChC) density, and associated phagocytic and inflammatory cells, as described.^7^ Near the GA border, where retina was attached, we also measured the thickness of OPL–ELM, ONL, and inner segment myoid. When an assessment location had concurrently applicable phenotypes in one given layer, the most advanced one was recorded.

Atrophic retina remained attached to the underlying support tissues in one area. Although torsion (twisting) around this attachment is possible, we reduced the impact of torsion on alignment by considering the spatial ordering of tissue features (superior to inferior and nasal to temporal) across scans and sections. We accounted for shrinkage of detached retina. Inner retina layers were not measured because they exhibited postmortem edema.

Data were compared with similar data from the fellow eye of this same patient, which had macular atrophy meeting criteria for cRORA in association with Type 3 neovascularization,^28^ to 13 donor eyes with histologically diagnosed GA^7^ (See **Table 1, Supplemental Digital Content 5**, http://links.lww.com/IAE/A958), and to an eye with macular atrophy due to mixed causes.^29^

### Statistics

*P* values <0.05 were considered significant. For clinical imaging, Student's *t*-test was used to compare mean ART function values and quality values between OCT scans with visible versus invisible ELM descent. Binary logistic regression was used to assess the influence of ART function and quality in visualizing the ELM descent. For histology, linear regression models were used to compare layer thicknesses and ChC density across the locations. Tissue samples were assumed to be independent of each other.

## Results

### Clinical Imaging

**Fig. 3.**
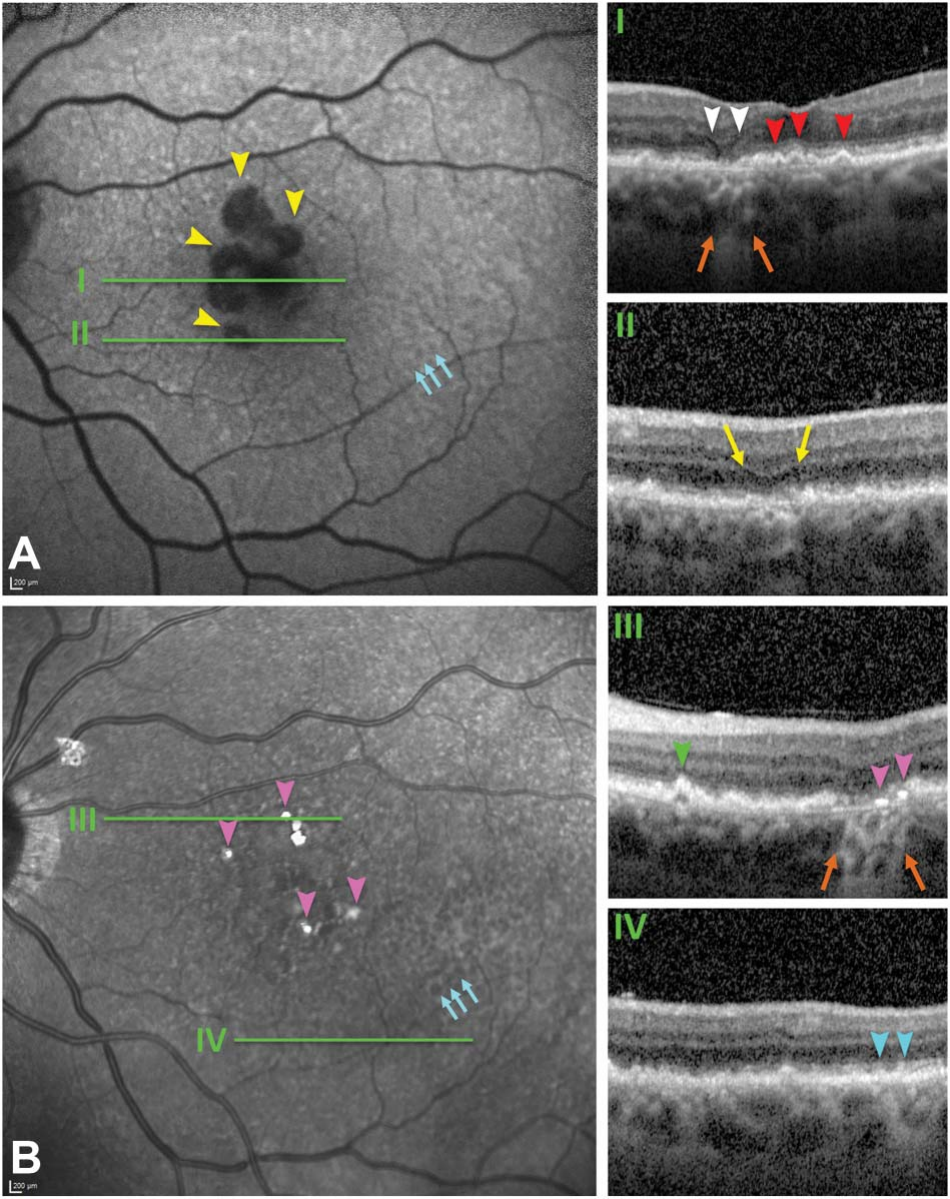
Main OCT features related to GA secondary to AMD. Green lines in (**A** and **B**) correspond with the OCT B-scans (**I**–**IV**). **A.** Fundus autofluorescence shows multilobular areas of hypoautofluorescence corresponding to RPE atrophy (yellow arrowheads) that lack hyperautofluorescent margins. Hypoautofluorescent dots concentrated temporally correspond to subretinal drusenoid deposits, (also called pseudodrusen; teal arrows) by correspondence with Panel B. **B.** Near-infrared reflectance demonstrates an area of several target-like pseudodrusen (dot or Type 3 SDD, teal arrows), a rough texture elsewhere in the macula, and intensely hyperreflective plaques (pink arrowheads). The atrophic area and refractile drusen are minimally visible. Optical coherence tomography B-scans at baseline (12 months before death; **I**, **II,** and **IV**) and at the last available visit (4 months before death, **III**). Orange arrows, hypertransmission, signifying RPE atrophy. **I.** Wedge-shaped hyporeflectivity (white arrowheads) at the GA border and RPE elevations with homogeneously reflective contents consistent with soft drusen (red arrowheads); (**II**) an area of nascent GA (yellow arrows)^69^ with diffuse thickening of the RPE–BLamD–Bruch membrane complex; (**III**) an area of GA (orange arrows) showing two focal hyperreflective lines above and parallel to the Bruch membrane (pink arrowheads). A pyramidal RPE elevation with hyperreflective dots and hyporeflective interior represents a refractile druse with calcific nodules (green arrowhead; Figure 4). **IV.** SDD (teal arrowheads). The ART (mean number of scans per image) ranged from 29 on (**I**) to 9 to 10 on (**II**–**IV**), and quality values ranged from 17 to 20 dB on (**II**–**IV**) to 28 dB on (**I**), accounting for differences in visualization of details.

Figure 3 shows multimodal in vivo clinical images. Baseline FAF highlights multilobular areas of marked hypoautofluorescence without distinct hyperautofluorescence at the margins^40^ (Figure 3A). Near-infrared reflectance did not clearly reveal the atrophic area or surrounding drusen but did show centrally located focal plaques of intense hyperreflectivity (Figure 3B). In the temporal perifovea, NIR showed a pattern of hyporeflective annuli surrounding an isoreflective core, consistent with target-appearance pseudodrusen^41^ (also called dot or Stage 3 SDD)^16,42^ (Figure 3B). These were just barely apparent in FAF (Figure 3A). In nontemporal perifoveal quadrants, both NIR and FAF showed a rough texture not resembling target pseudodrusen.

Optical coherence tomography B-scans (green lines in Figure 3) showed GA in the nasal parafovea, with characteristic hyporeflective wedge-shaped bands just inside the atrophic area and RBB thickening throughout the macula. Subfoveal mound-shaped RPE elevations with homogeneous and mildly hyperreflective contents represented soft drusen (Figure 3I, red arrowheads). Areas of incomplete RORA (iRORA) were associated with diffuse thickening of the RBB (Figure 3II, yellow arrows). Refractile drusen were identified by pyramidal shape, hyperreflective dots, and hyporeflective interior (Figure 3III, green arrowhead). Subretinal drusenoid deposits exhibiting characteristic hyperreflectivity internal to a minimally perturbed RBB were multiple, widely distributed, and distinguishable from drusen (Figure 3IV, teal arrowheads). The choroid had relatively preserved thickness and clinical appearance. No pachyvessels,^43^ hyporeflective caverns,^44^ fibrosis, or neovascularization were detected.

**Fig. 4.**
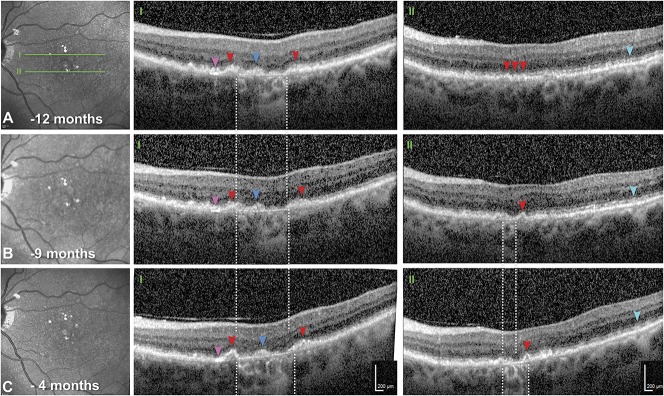
Eye-tracked OCT scans showing progression of GA. **A**–**C.** Near-infrared reflectance at 12, 9, and 4 months before death. Green lines (**A**) reference OCT B-scans at 2 different planes (**I** and **II**). **I** and **II.** White-dashed lines delimit choroidal hypertransmission, signifying GA. In both scans, the choroid is thick and lacking abnormalities in either vessel caliber or overall appearance (i.e., no pachyvessels or infiltrative or degenerative changes). The ART function was 9 to 10. Quality values ranged from 17 to 24 dB. **I.** Geographic atrophy expands from baseline. Retinal pigment epithelium elevations with smooth contours and homogeneously hyperreflective contents (red arrowheads) are consistent with soft drusen. Above and parallel to the Bruch membrane is a focal, intensely hyperreflective line (pink arrowheads), and hyperreflective material persists in the atrophic area (blue arrowheads). **II.** Three months after baseline, a new area of GA appears. At baseline and throughout follow-up, RPE elevations consistent with soft drusen (red arrowheads) are present. Hyperreflective material above the RPE, sometimes regularly spaced, represents widespread SDDs (teal arrowheads).

During 8 months of follow-up involving 6 clinic visits, areas of iRORA and GA expanded (1.27–1.85 mm^2^, +146%) and merged (Figure 4I, A–C; See **Figure 2, Supplemental Digital Content 2**, http://links.lww.com/IAE/A955). These areas exhibited loss of outer retinal bands (ONL, ELM, ellipsoid zone, and interdigitation zone), discontinuation of the RPE-BL band that uncovered BrM, and then choroidal hypertransmission. Also, RBB thickened at the GA edge (Figure 4, IA, red arrowheads). One area with soft drusen at baseline (Figure 4, IIA) underwent progressive subsidence of inner nuclear and outer retinal layers, culminating as new GA (Figure 4, IIB and C). Within the temporal area of clearly visible SDD, RBB thickness was 22.88 ± 6.4 *µ*m. Elsewhere in the perifovea, where SDD could not be measured with confidence, RBB thickness was 47.41 ± 17.09 *µ*m (2.0 mm nasal) and 31.77 ± 14.22 (2.0 mm temporal). At the GA border (Table 1), the RBB progressively thickened toward the ELM descent at Visits 1 and 2, but did not thicken between visits. Subfoveal choroidal thickness was 270 *µ*m and 247 *µ*m at Visits 1 and 2, respectively.

**Table 1.**
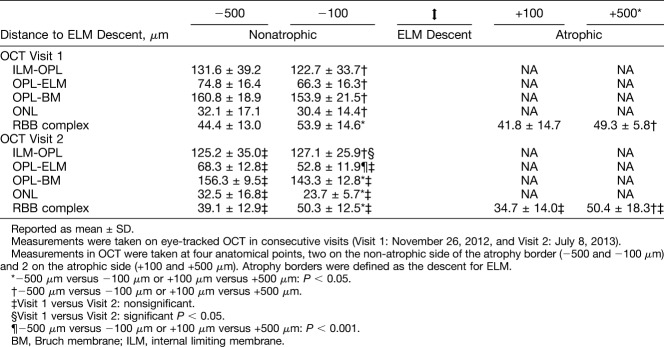
Quantitative Analysis Using OCT

In the 6 available OCT volumes, the ELM descent was detectable in 8 to 9 of 10 B-scans crossing GA in 3 volumes, 2 to 3 of 10 scans in 2 volumes, and in no scans in one volume. Automatic real time function and quality values ranged from 7 to 11 and 3 to 27, respectively. Relative to B-scans with visible ELM descents, B-scans with invisible ELM descents had similar ART values (8.83 vs. 8.89; *P* = 0.960) and significantly lower-quality values (17.81 vs. 12.96 dB; *P* = 0.001).

### Histology of the Retinal Pigment Epithelium–Deposit Complex

**Fig. 5.**
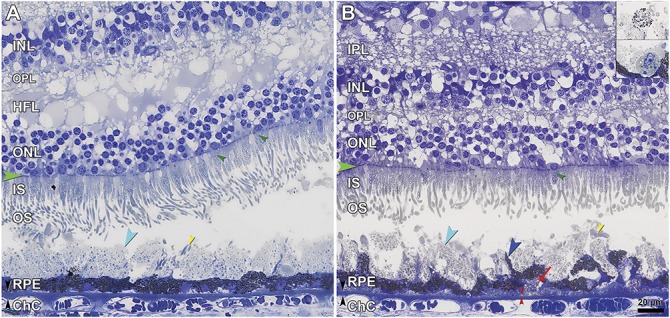
Subretinal drusenoid deposits (SDD) and surrounding cells correlating to different appearances in multimodal imaging. IPL, inner plexiform layer; INL, inner nuclear layer; OPL, outer plexiform layer; IS, inner segments; OS, outer segments; RPE, retinal pigment epithelium; ChC, choriocapillaris. ELM, green arrowheads; SDD, aqua arrowheads; Bruch membrane, black arrowheads. Retracted mitochondria in cone IS, dark green arrowheads. **A.** In the temporal area with target-like pseudodrusen (Figure 3, A and B), SDD (aqua arrowhead) are confluent and punctuated with OS crossing the deposit to reach RPE (yellow arrowheads), which is nonuniform in thickness. **B.** In areas with a roughened texture on NIR imaging (Figure 3B), SDD (aqua arrowhead) is less confluent with some caps resembling OS (yellow arrowhead), OS are shorter, and RPE is continuous but markedly dysmorphic with inward extensions (blue arrowhead). Basal linear deposit, red arrowheads. Basal mound, red arrow. Insets: top, sloughed^70^ RPE cells. Bottom, phagocytes occasionally appearing among SDD in other sections. Detached retina in B was digitally reapproximated.

**Fig. 6.**
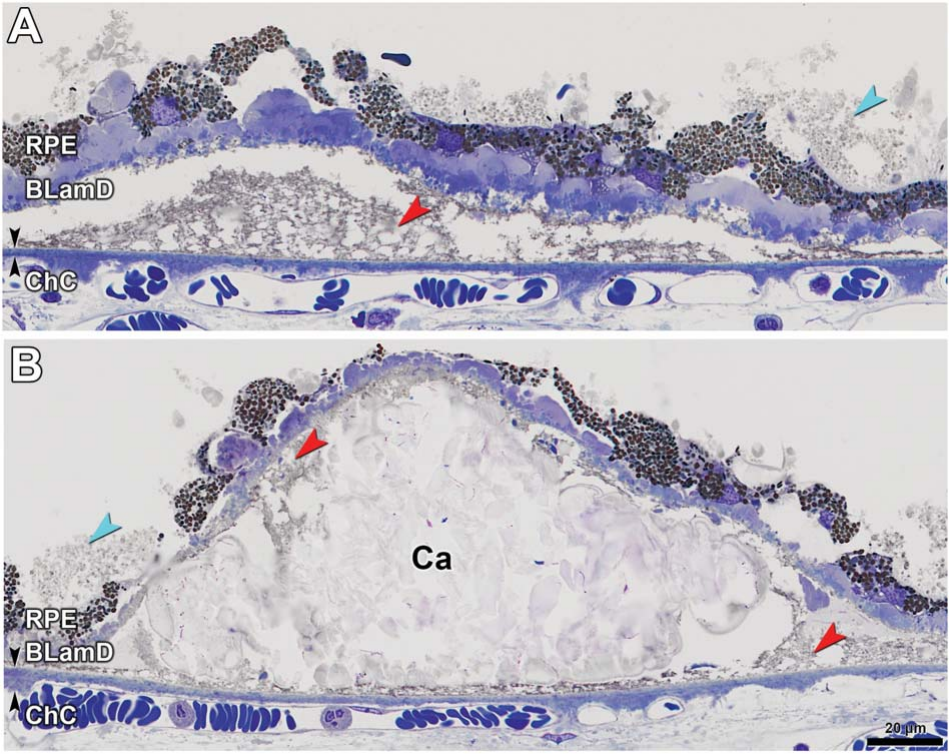
Soft and calcified drusen in GA secondary to AMD. Bruch membrane, black arrowheads. Lipid-rich lipoprotein-derived debris (membranous debris of Sarks), red arrowheads. Subretinal drusenoid deposit, aqua arrowheads. RPE, retinal pigment epithelium; BLamD, basal laminar deposit; ChC, choriocapillaris. **A.** Soft druse with partially preserved contents has late (scalloped) BLamD. **B.** Calcific nodules (Ca) are one end-stage of soft drusen. Some original contents still remain.

**Fig. 7.**
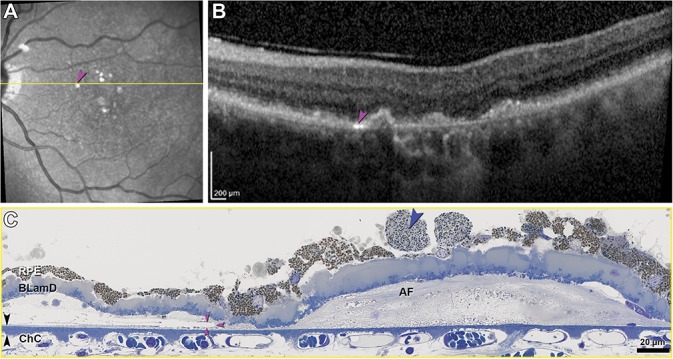
Cholesterol clefts, avascular fibrosis, and dysmorphia of RPE. RPE, retinal pigment epithelium; BLamD, basal laminar deposit; ChC, choriocapillaris; AF, avascular fibrosis; Bruch membrane, black arrowheads. **A.** Near-infrared reflectance imaging shows multiple hyperreflective plaques (pink arrowhead). Yellow line, B-scan level in (**B**). **B.** An intensely hyperreflective line parallels BrM (pink arrowhead). Adjacent is an irregular RPE elevation with moderate, heterogeneous reflectivity distinct from a soft druse. **C.** A cholesterol cleft (pink arrowheads) correlates to the hyperreflective line in (**A**)/(**B**). The distance from top and bottom surfaces of the cleft to the inner surface of BrM is 5.1 and 1.8 *µ*m, respectively. Sloughed RPE cells^70^ (blue arrowhead) have smaller, more loosely packed, and greener-staining granules than adjacent dysmorphic yet still continuous RPE.

**Fig. 8.**
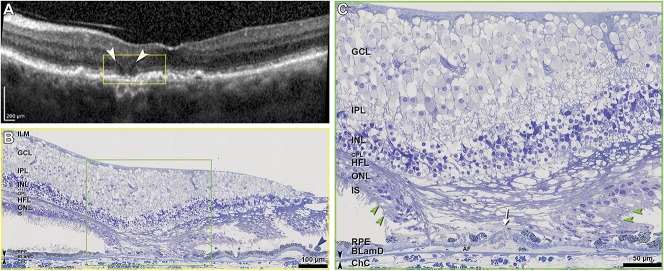
In vivo OCT and histopathology of Monés hyporeflective wedge in GA. ILM, inner limiting membrane; GCL, ganglion cell layer; IPL, inner plexiform layer; INL, inner nuclear layer; OPL, outer plexiform layer; HFL, Henle fiber layer; ONL, outer nuclear layer; IS, inner segments, RPE, retinal pigment epithelium; BLamD, basal laminar deposit; ChC, choriocapillaris; descent of the ELM, green arrowheads; Bruch membrane, double black arrowheads. **A.** Optical coherence tomography shows two hyporeflective wedged-shaped bands (white arrowheads), with overlying subsidence of INL, interrupted ELM (descents are not visible), interrupted ellipsoid zone, and RPE elevations. Single black arrowhead, sloughed RPE. **B** and **C.** Histology shows ordered HFL (i.e., parallel fibers, despite artifactual postmortem separation), and no cellular infiltration degenerating photoreceptors, and atrophic ONL. Müller cell processes invade the sub–RPE-BLamD space (white arrow). AF, avascular fibrosis.

**Fig. 9.**
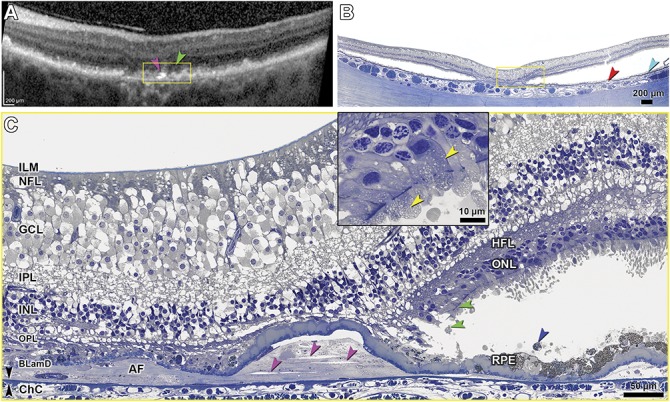
In vivo OCT and histopathology correlation of photoreceptor degeneration in GA. ILM, inner limiting membrane; GCL, ganglion cell layer; IPL, inner plexiform layer; INL, inner nuclear layer; OPL, outer plexiform layer; BLamD, basal laminar deposit; ChC, choriocapillaris. Bruch membrane, black arrowheads. **A.** In vivo OCT shows cRORA, OPL subsidence, inner retina thickening, and intensely hyperreflective lines (pink arrowhead) above BrM. **B.** Correlative histology shows atrophy of photoreceptor and RPE layers flanked by artifactual postmortem retinal detachment. Drusen, red arrowhead; SDD, teal arrowhead. **C.** In the yellow-framed atrophic area of Panels A and B, the ONL and RPE are absent, and avascular fibrosis (AF) is seen beneath BLamD. Clefts in the AF (pink arrowheads) correlate to the hyperreflective lines in panel A. Distances from the top-bottom surfaces of the clefts (pink arrowheads) to the inner collagenous layer of BrM are (from left to right) 11.8 to 10.2 *µ*m, 30.4 to 26.8 *µ*m, and 23.8 to 19.9 *µ*m, respectively. In the nonatrophic area, near the descent of ELM (green arrowheads), photoreceptor nuclei and mitochondria (yellow arrowheads, inset) are retracted toward HFL. Sloughed RPE, blue arrowhead. At the upper right is artifactual thickening of inner retina due to edema (See **Figure S1, Supplemental Digital Content 1**, http://links.lww.com/IAE/A954).

Geographic atrophy corresponded to a small, multilobular atrophic area in the nasal parafovea, 408 to 882 *µ*m from the foveal center. Figures 5–9 correlate point-by-point ex vivo histology to in vivo clinical imaging. Table 2 and **Supplemental Digital Contents 6–8** (See **Tables 2–4**, http://links.lww.com/IAE/A959, http://links.lww.com/IAE/A960, http://links.lww.com/IAE/A961) show cellular phenotype frequencies and layer thicknesses, for RPE–deposit complex, BrM–ChC, and outer retina, in order. To clarify relations with both photoreceptor topography and GA borders, Table 2 shows all RPE–deposit complex thicknesses, from nasal to temporal, across the histological sections.

**Table 2.**
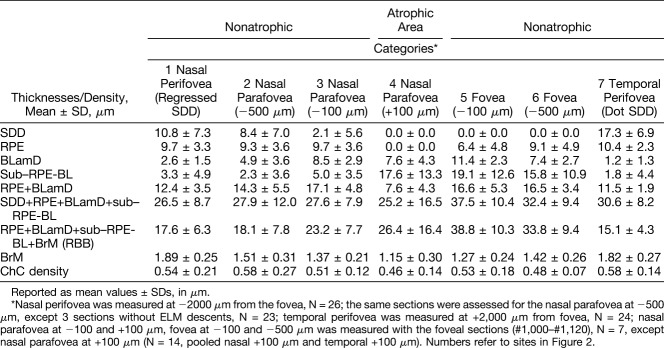
Topography of Multilayer AMD Pathology in the Index Case

Subretinal drusenoid deposit was widespread in the macula and beyond, yet absent in and near the atrophic areas and over subfoveal drusen (Figure 5 and Table 2). In 45 sections (mean length 7.35 ± 0.65 mm), coverage of macular RPE by SDD overall was 76.9% ± 9.9%. Because SDD was clearly visible in NIR only in temporal macula (Figures 3–4), we compared eccentricity-matched areas in temporal and nasal perifovea (Figure 5 and Table 2). In both places and consistent with previous reports,^13,22,45,46^ SDD comprised mounds of extracellular material consisting of a dispersed phase of small, regularly spaced, and gray-staining particles within a flocculent and light-staining continuous phase. Some deposits had caps of outer segment fragments (Figure 5B, yellow arrowhead). Infrequently, sloughed RPE and non-RPE cells were also present (Figure 5B, insets). In temporal perifovea, continuous SDD overlaid continuous RPE, punctuated by fascicles of RPE apical processes containing melanosomes and extending to contact outer segments (Figure 5A). Relative to temporal SDD (Figure 5A), nasal SDD (Figure 5B) was thinner, less intact, and flanked by RPE with apically projecting, organelle-filled extensions of cell bodies, sometimes bizarrely shaped, which embraced the deposits. Nasal SDD was also associated with significantly thicker BLamD (Table 2) and overlying photoreceptors with shorter outer segments (Figure 5B). At the nasal GA border, SDD thinned (Table 2).

Corresponding to subfoveal soft drusen on OCT (Figure 3I, red arrowheads) were RPE elevations containing a homogeneous, brown-stained material.^47^ Retinal pigment epithelium elevations containing similar material were found throughout the nonatrophic area of all sections and were continuous in the sub–RPE-BL space with BLinD. One soft druse end-stage is multilobular calcific nodules that replace the original lipid-rich contents in the sub–RPE-BL space,^19^ and these were common (18/45 sections). A histologic druse with nodules (Figure 6B) correlated to a druse with a hyporeflective interior on in vivo OCT in Figure 3III (green arrowhead).

In the sub–RPE-BL space of GA (30/45 sections) and extending beyond it under the fovea was a fibrotic material of undulating thickness, with few cells and no apparent vessels (avascular fibrosis, Figures 7–9). This fibrosis was continuous with soft drusen/BLinD, and individual RPE elevations could contain both materials (red arrowheads; See **Figure 3, Supplemental Digital Content 3**, http://links.lww.com/IAE/A956, showing contents of sub–RPE-BL space). Within fibrosis and not within soft drusen/BLinD were found clefts created by the extraction of cholesterol crystals by solvents during histological processing (Figures 7C and 9C). These correlated to hyperreflective lines above and parallel to BrM on OCT (Figures 7B and 8A, pink arrowheads) and intensely reflective plaques on NIR imaging (Figure 7A, pink arrowhead). In the sub–RPE-BL space in atrophic and nonatrophic areas were phagocytes, multinucleated giant cells, and subducted RPE (See **Figure 4, Supplemental Digital Content 4**, http://links.lww.com/IAE/A957). In the sub–RPE-BL space of the atrophic area (26/45 sections) were processes (Figure 9C, white arrow) resembling those of similar size and color internal to persistent BLamD in this and other GA eyes and considered Müller cells.^7^

Considering BLamD and RPE, BLamD was thick and present in early (palisade) and late (scalloped) forms in the nonatrophic area (Figures 6A, B, and 7C). Basal laminar deposit was persistent across GA but thinner (Figures 8C and 9C). In GA, more than half of sampled locations had dissociated RPE (Figures 8C and 9C). In nonatrophic areas, sloughed RPE in the subretinal space (Figures 7C and 8C, blue arrowhead) were hypertrophic and spherical, with moderately concentrated, greenish-stained lipofuscin and melanolipofuscin granules (Figure 7C). In severely nonuniform but still epithelial RPE in the same histologic section, granules were packed and stained bronze, indicating molecular differences between cells in these two states. Retinal pigment epithelium and RPE + BLamD thickened significantly (*P* < 0.0001) across the nonatrophic area toward the ELM descent, then declined on the atrophic side (Table 2). Near GA, sloughed RPE and individual melanosome/lipofuscin granules were found near the ELM descent only (See **Table 2, Supplemental Digital Content 6**, http://links.lww.com/IAE/A959), and no intraretinal RPE cells were detected.

### Histology of the BrM, Choriocapillaris, and Choroid

BrM was notable for the absence of refractile unstained patches that signify calcification. As shown in Table 2, BrM was thick in the perifovea (1.89 ± 0.25, 1.82 ± 0.27 *µ*m) and thinned across the ELM descent into GA (1.77 ± 0.27, 1.58 ± 0.38, 1.40 ± 0.23 *µ*m; *P* = 0.0053). Figures 6B, 7C, 8C, and 9C show relatively intact ChC in nonatrophic and atrophic areas, with few retracted capillaries or ghost capillaries. Choriocapillaris density was similar at approximately 0.60 in the perifovea and parafovea (Table 2) and across ELM descent into GA (Table 2). The frequency of unremarkable ChC was high throughout, and depillared BrM was not detected (See **Table 3, Supplemental Digital Content 7**, http://links.lww.com/IAE/A960). Corresponding to the OCT B-scans, the choroid was found to have relatively preserved thickness (Figure 8, A and B). The stroma was edematous, and large vessels contained blood, likely due to fixation by infusion.^28^ Friedman lipid globules^44^ were common in the choroid and in sclera (42/45 and 20/45 sections, respectively; not shown).

### Histology of Neurosensory Retina

By both in vivo OCT and ex vivo histology, the atrophic areas (Figures 8 and 9) had ELM descents at the nasal and temporal aspects, with subsidence of OPL and inner nuclear layer between. External limiting membrane descents delimited the atrophic area (Figure 9C, green arrowheads), and between them, the ONL was completely atrophic, and there was no ELM. Optical coherence tomography shows hyporeflective wedges^48^ on the atrophic sides of each ELM descent (Figure 8A). Histology (Figure 8, B and C) revealed Henle fiber layer (HFL) that is ordered (i.e., parallel fibers), despite artifactual separation of individual fibers, and lacking cellular infiltration (Figure 8C). Internal to the wedge, the inner nuclear layer sagged downward (Figure 8B). On the nonatrophic side of the ELM descent (Figure 9, A–C) were a loss of outer segments, progressive shortening of inner segments, and inward translocation of mitochondria toward the cell body. Dyslamination of HFL/ONL and outer retinal tubulation/photoreceptor islands, two severe forms of photoreceptor degeneration and gliosis,^7^ were not detected in this eye.

Photoreceptor layer phenotypes and thicknesses are shown in **Supplemental Digital Content 8** (see **Table 4**, http://links.lww.com/IAE/A961). Ectopic photoreceptor nuclei in OPL/HFL were common at the nonatrophic side of the ELM descent, as were ectopic photoreceptor nuclei in the inner segment. Five measures of photoreceptor abundance and health declined significantly (*P* < 0.0001) to zero on the atrophic side of the ELM descent (frequency of unremarkable ONL, frequency of continuous ONL, ONL thickness, rows of ONL nuclei, and inner segment myoid thickness). Absence of continuous ONL, ELM, and RPE layers (Figure 8C) correlates to GA (Figure 8A) on OCT.

## Discussion

We build on foundational clinicopathologic correlation with panoramic electron microscopy by J.P. and S.H. Sarks and M. C. Killingsworth and a comprehensive OCT catalog by Fleckenstein et al.^49^ Our index case of GA secondary to AMD had a typical clinical presentation. The index case informs on the extent, topography, and end-stages of extracellular deposits central to AMD progression,^50^ photoreceptor depletion and gliosis, and the detection limits of current clinical imaging. Data are compared to other donor eyes with GA and clinicopathologic correlation of macular atrophy associated with neovascularization.^7,28,29^

Mound-shaped RPE elevations with homogeneous and mildly hyperreflective contents seen in OCT B-scans correlated with histologically identified soft drusen containing a homogeneous lipid-rich material–lacking cells and cellular fragments.^17^ Contents were interpreted as partly preserved membranous debris of Sarks et al,^19^ which itself is partly preserved masses of lipoprotein particles, both native and fused.^47^ Much evidence supports the idea that the RPE constitutively secretes large (∼80-nm diameter) apolipoprotein B, E-containing lipoprotein particles rich in esterified cholesterol that fill BrM throughout adulthood to form BLinD and soft drusen, as egress across the RPE–BrM–ChC complex is impaired, age-dependently.^51,52^ Very large drusen have a lifecycle of growth due to underclearance of these normally secreted RPE products and collapse after RPE migration and death terminates their production.^23,46^

Of soft druse end-stages described by the Sarks et al^19^ (reduced production, removal by macrophages, Müller cell processes penetrating BLamD, calcification, and replacement by collagen fibers), the index case exhibited the latter four, with calcification and fibrotic change visible by OCT. Within numerous RPE elevations were nodules, i.e., multilobed refractile structures 5 to 100 *µ*m in diameter that are rich in a molecularly distinct hydroxyapatite. Nodules were recently confirmed as correlating to a heterogeneous internal reflectivity of drusen associated with 6-fold increased risk of progression to advanced disease.^53,54^ As seen by the Sarks, avascular fibrosis distinct from both fibrin deposition and exudation-associated fibrosis replaced soft drusen/BLinD while maintaining the shapes of drusenoid RPE elevations.^55,56^ We saw histologic evidence without imaging correlates for sub–RPE-BL cells potentially clearing drusen (Müller cells,^7,20,57^ subducted RPE, and probable macrophages).^58^

Within avascular fibrosis were single or sparse cholesterol crystals that could be correlated to intensely reflective areas in NIR and reflective lines in some OCT scans (Figures 3, 7, and 9, pink arrowheads). Fleckenstein et al^49^ and others^59^ attributed reflective horizontally oriented plaques to “densification” of BrM (e.g., electron density on transmission electron microscopy).^19^ Querques et al^60^ proposed that hyperreflective, variably oriented lines in regressing drusen resulted from either BrM splitting and bowing inward or a process “similar to the onion sign.”^61^ We subsequently correlated the onion sign in eyes with sub–RPE-BL hemorrhage and fluid to groups of cholesterol crystals,^36^ suggesting that an aqueous environment is required for super-saturation and precipitation. Conversion of the sub–RPE-BL environment from primarily lipid (soft drusen/BLinD) to nonlipidic (hemorrhage, fluid, and fibrosis) may promote cholesterol crystallization in the available fluid, e.g., the hydration water of collagen fibrils. Our data thus support crystallization as a biomarker for the replacement of soft drusen contents, while also not supporting BrM as a source of mirror-like reflectivity, because BrM in the index case lacked calcification. The Fleckenstein plaques^49^ may thus represent cholesterol crystals that are too close to BrM (<1.8–11.8 *µ*m) to be resolved with present technology. Conversely, RPE elevations over avascular fibrosis can be distinguished from dome-shaped soft drusen by the slight irregularity of contour and content of reflective crystals (Figure 3).

Subretinal drusenoid deposit was extensive, thick, acellular, and undetectable under the fovea, consistent with a topography resembling that of rod photoreceptors.^62^ Solid extracellular deposits between photoreceptors and RPE account for reticular pseudodrusen, as established by direct clinicopathologic correlation,^14,15^ histological survey,^13,45^ and clinical OCT.^16,63^ In the index case, histologically detectable SDD was far more extensive than that seen in vivo. The rough texture seen on NIR and FAF (Figure 3, A and B) may thus signify confluent and degenerate^25^ SDD (Figure 5B). Building on our proposal that SDD entails dysregulation of the same lipid cycling system that produces drusen,^13,52^ we suggest that SDDs signify both progression risk and some functionality of participatory photoreceptors, RPE, and Müller glia. Subretinal drusenoid deposits also exhibit growth and regression.^25^ Without an obvious barrier to transport such as BrM, SDD may dissipate because of underproduction by these cells, as they degenerate. Subretinal drusenoid deposit has been misclassified as soft drusen or omitted from grading systems using color fundus photography;^27^ yet, photoreceptor degeneration and a distinctive RPE dysmorphia associated with SDD underscore its place in AMD.

In the RPE layer, the index case is representative of the appearance and enlargement of GA, in the absence of neovascular findings. The index eye exhibited thickening of the RBB complex toward the ELM descent in OCT and histology (Table 2). This thickening is accounted for by progressive RPE dysmorphia atop a BLamD of relatively uniform thickness. Retinal pigment epithelium dysmorphia at the ELM descent was first described by the Sarks and recently quantified in donor eyes^8,39^ and in direct clinicopathologic correlation of macular atrophy.^29^ In the index eye, the retina remained attached at the atrophic area, a common finding in donor eyes with GA, possibly due to Müller cell interaction with persistent BLamD^18^ that may or may not itself be visible. The hyporeflective wedge of Monés^48^ is a reliable OCT signature in GA, sometimes visible internal to the entire border in *en face* OCT. Despite suboptimal tissue preservation, Müller fibers in the HFL remained parallel and ordered, without cellular infiltration, consistent with permissiveness to transmitted light and low reflectivity typical of a wedge. By contrast, punctate reflectivity^20^ in atrophic areas may result from disordered Müller processes, possibly predictive of atrophy expansion.^64^

Compared to similarly analyzed donor eyes with GA,^7^ the index eye differed by having undetectable HFL/ONL dyslamination or intraretinal RPE, relatively healthy ChC, noncalcified BrM, and prominent avascular fibrosis. Relative to the comparison cases, the index eye had a small multilobular atrophic area (See **Table 1, Supplemental Digital Content 5**, http://links.lww.com/IAE/A958), suggesting that a relatively recent onset of atrophy could underlie these differences. Like the fellow eye that progressed to Type 3 neovascularization,^28^ the index eye also had abundant extracellular deposits, relatively intact ChC, cells in the sub–RPE-BL space, and progression to advanced AMD at the same (parafoveal) eccentricity, supporting a causal relationship with the topography of outer retinal cells.^62,65^ However, although the index eye had sub–RPE-BL avascular fibrosis, in the fellow eye, Müller cell processes accompanied a neovascular stalk from the retina that implanted in this compartment.

We provide new information about device-independent and device-dependent visibility of specific pathologies. Both the atrophic zone and drusen were poorly visible in the NIR locator image. Light reflected from the sclera impacts the intensity of retinal reflectivity^66^ and autofluorescence,^67^ and eyes with thick choroids, like the index eye, show low NIR within areas of GA. Although BLamD was >11-*µ*m thick under the fovea (Table 2), it could not be cleanly delimited from the RPE layer in OCT. The index eye had notably little focal hyperautofluorescence at the atrophy margin in baseline FAF and accordingly had relatively few sloughed and intraretinal RPE on histology 12 months later. Numerous histologic lipid globules did not manifest as numerous hyporeflective caverns^44^ because caverns are best seen in en face reconstruction of dense OCT B-scan raster patterns.

Our data help define imaging parameters necessary to consistently visualize the ELM descent, proposed as a histologically meaningful border of atrophy.^7,8,39^ Descents were visible in a scan with ART = 29 and quality = 24 dB (Figures 3-I) but not in scans with ART = 7 to 11 and quality = 11 to 19 dB (Figure 4, I and II). Separately, we showed that in macular atrophy, 100% of ELM descents were detected, with ART of 7 and quality of 33 dB.^29^ The invisibility of the ELM descent in color fundus photography and FAF imaging, in concert with inaccurate histologic quantification of outer retinal cells in GA,^68^ can lead to an overestimate of viable retina available for therapeutic rescue in the atrophic zone. The loss of relevant detail at quality ≤15 dB when ART is below 9 to 10 encourages heightened attention to these settings for accurate assessment of GA and its precursors.

Strengths of this study include serial eye-tracked OCT with the last clinic visit 4 months before tissue recovery, baseline FAF imaging, a detailed and comprehensive histology technique, multiscale viewing of both digital sections and OCT scans, and current nomenclature for both OCT and AMD pathology. Limitations include insufficient image quality in OCT volumes to reveal all relevant features, poor preservation of druse contents, and postmortem detachment that impeded comprehensive quantification of retinal morphology. Nevertheless, this is the first clinicopathologic correlation of GA with in vivo OCT, NIR, and FAF imaging, with the most complete accounting of currently recognized AMD layers since the Sarks' monumental description. Age-related macular degeneration's notorious complexity has been exacerbated by limitations of imaging technologies that could not reveal major pathology within and beyond the RPE layer. The catalog of histologically validated OCT signatures, expanded by this report, can serve as references for other modalities. A timeline from AMD precursors to end-stages visible clinically at the subcellular level is a holy grail for multifactorial diseases of aging. Recent trial imaging data can be interpreted to inform preventive measures and direct new therapies to disease stages before irreversible tissue damage.

## Supplementary Material

SUPPLEMENTARY MATERIAL
