# Supplementary material for: CLINICOPATHOLOGIC CORRELATION OF GEOGRAPHIC ATROPHY SECONDARY TO AGE-RELATED MACULAR DEGENERATION
Source: Retina. 2019 Feb 6;39(4):802–16. doi: 10.1097/IAE.0000000000002461 (PMC6445604; doi:10.1097/IAE.0000000000002461)
Supplement: SUPPLEMENTARY MATERIAL [file retina-39-802-s001.pdf]

Supplementary Figure 1: In vivo and ex vivo near infrared reflectance (NIR) and optical coherence tomography (OCT) B-scans.

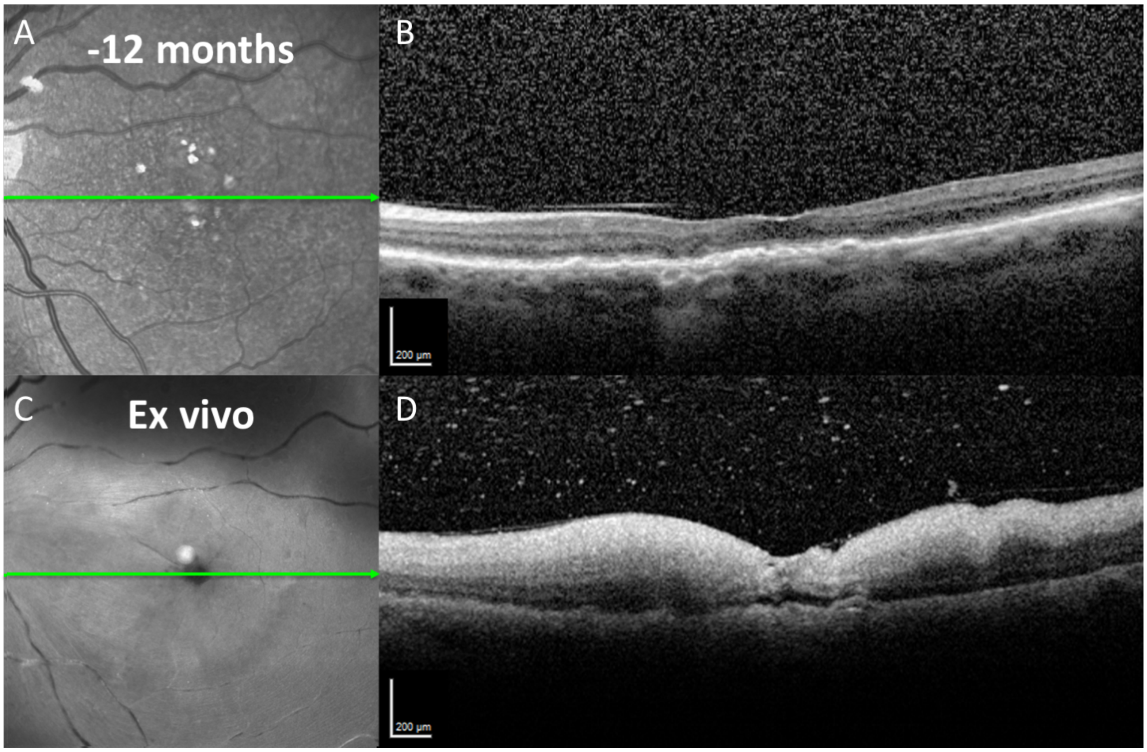

A. Near infrared reflectance (NIR) at 12 months before death showing the presence of multiple subretinal drusenoid deposits. **B.** OCT B-scan over the area shown on A (green arrow) showing an area of retinal pigment epithelium (RPE) and outer retinal atrophy associated with thickening of the RPE-Basal Laminar Deposit-Bruch's membrane complex. C. *Ex vivo* NIR image. **D.** *Ex vivo* OCT B-scan over the area referenced on C (green arrow) showing increased reflectivity of the inner retinal layers with marked posterior shadowing due to post-mortem edema of inner retina, which distorts layers temporal to the fovea. RPE-BLamD thickening is also evident in the subfoveal region.
