# Supplementary material for: CLINICOPATHOLOGIC CORRELATION OF GEOGRAPHIC ATROPHY SECONDARY TO AGE-RELATED MACULAR DEGENERATION
Source: Retina. 2019 Feb 6;39(4):802–16. doi: 10.1097/IAE.0000000000002461 (PMC6445604; doi:10.1097/IAE.0000000000002461)
Supplement: SUPPLEMENTARY MATERIAL [file retina-39-802-s002.pdf]

Supplementary Figure 2. Changes over time in the area of geographic atrophy (GA).

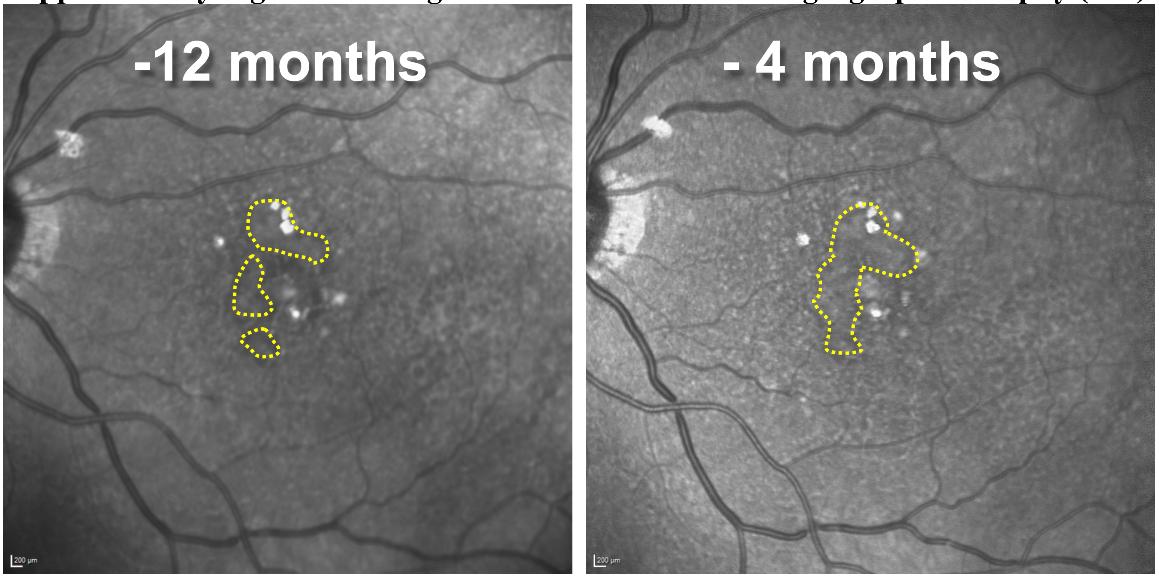

Near infrared reflectance at 12 (A) and 4 months (B) before death. Growing of the areas of GA is observed (yellow-dashed area) associated with the presence of diffusely distributed subretinal drusenoid deposits.
