# Supplementary material for: CLINICOPATHOLOGIC CORRELATION OF GEOGRAPHIC ATROPHY SECONDARY TO AGE-RELATED MACULAR DEGENERATION
Source: Retina. 2019 Feb 6;39(4):802–16. doi: 10.1097/IAE.0000000000002461 (PMC6445604; doi:10.1097/IAE.0000000000002461)
Supplement: SUPPLEMENTARY MATERIAL [file retina-39-802-s003.pdf]

Supplementary Figure 3. Continuity of avascular fibrosis (AF) with soft drusen material, in the sub-RPE-basal lamina (sub-RPE-BL) space.

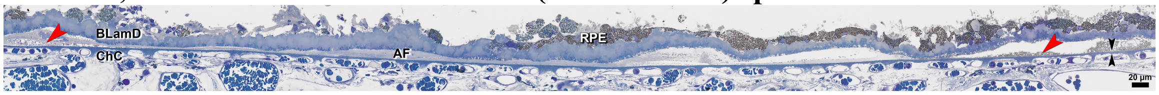

Epoxy resin sections of the osmium tannic acid paraphenylenediamine-post-fixed specimen, stained with toluidine blue. RPE, retinal pigment epithelium; BLamD, basal laminar deposit; Bruch's membrane, black arrowheads; ChC, choriocapillaris. Avascular fibrosis is present in the sub-RPE-BL space inside and around the atrophic area, and is continuous with basal linear deposit (red arrowheads).
