# Supplementary material for: CLINICOPATHOLOGIC CORRELATION OF GEOGRAPHIC ATROPHY SECONDARY TO AGE-RELATED MACULAR DEGENERATION
Source: Retina. 2019 Feb 6;39(4):802–16. doi: 10.1097/IAE.0000000000002461 (PMC6445604; doi:10.1097/IAE.0000000000002461)
Supplement: SUPPLEMENTARY MATERIAL [file retina-39-802-s004.pdf]

Supplementary Figure 4. Cells and avascular fibrosis (AF) in the sub-RPE-basal lamina (sub-RPE-BL) space.

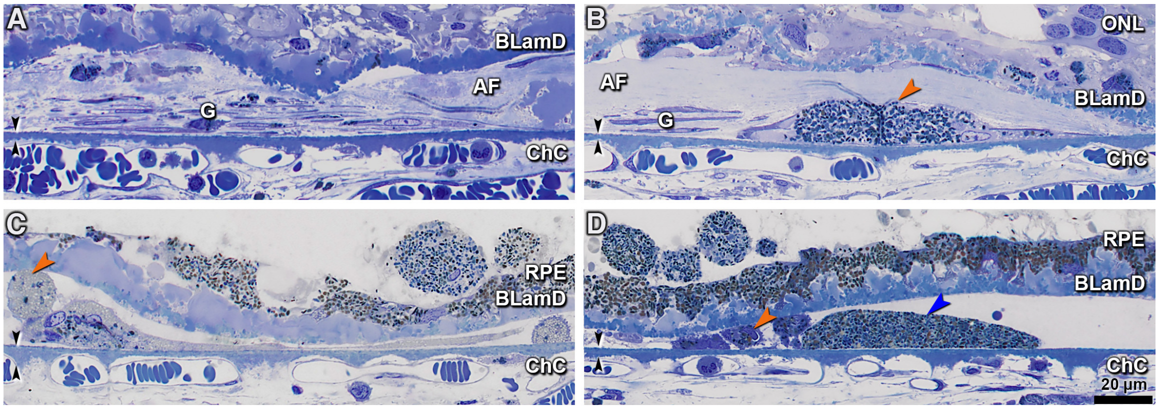

ONL, outer nuclear layer; RPE, retinal pigment epithelium; BLamD, basal laminar deposit; Bruch's membrane, black arrowheads; ChC, choriocapillaris. Epoxy resin sections of the osmium tannic acid paraphenylenediamine-post-fixed specimen, stained with toluidine blue. Bar in (D) applies to all 4 panels. **A.** A multinucleated giant cell (G) and AF are between BLamD and inner collagenous layer of Bruch's membrane (sub-RPE-BL space) in the atrophic area. **B.** A macrophage-like cell with melanosome/lipofuscin granules (orange arrowhead), a multinucleated giant cell (G), and AF are in the atrophic area. **C.** Macrophages with lipid and melanosome/lipofuscin granules (orange arrowhead) are present in non-atrophic area. The RPE layer is partly intact. **D.** Purple-staining phagocytes with inclusions of melanosome/lipofuscin granules (orange arrowhead), a large subducted RPE cell with typical RPE granules (blue arrowhead) are present in non-atrophic area.
