# Supplementary material for: CLINICOPATHOLOGIC CORRELATION OF GEOGRAPHIC ATROPHY SECONDARY TO AGE-RELATED MACULAR DEGENERATION
Source: Retina. 2019 Feb 6;39(4):802–16. doi: 10.1097/IAE.0000000000002461 (PMC6445604; doi:10.1097/IAE.0000000000002461)
Supplement: SUPPLEMENTARY MATERIAL [file retina-39-802-s005.pdf]

Table S1. Atrophic length at fovea sections from GA database and the study eye.

GA border type	Total atrophic length (µm)	
	Absence of continuous RPE	Distance between ELM descents
Multilobular	4694	4586
Multilobular	4246	4131
Multilobular	3245	3257
Multilobular	3137	3157
Multilobular	2821	2744
Multilobular	1347	1400
Multilobular	1196	1011
Multilobular	**1127**	**965**
Unilobular	1650	1528
Unilobular	1040	679
Unilobular	1025	1038
Unilobular	891	891
Unilobular	709	691
Unilobular	525	301

Lengths in bold are measured from the study eye.
Mean ± SD (µm): multilobular-2727 ± 1385 (absence of continuous RPE), 2656 ± 1397 (distance between ELM descents); unilobular-973 ± 385 (absence of continuous RPE), 855 ± 413 (distance between ELM descents).
