# Supplementary material for: CLINICOPATHOLOGIC CORRELATION OF GEOGRAPHIC ATROPHY SECONDARY TO AGE-RELATED MACULAR DEGENERATION
Source: Retina. 2019 Feb 6;39(4):802–16. doi: 10.1097/IAE.0000000000002461 (PMC6445604; doi:10.1097/IAE.0000000000002461)
Supplement: SUPPLEMENTARY MATERIAL [file retina-39-802-s006.pdf]

Supplementary Table 2. RPE phenotypes and melanosome/lipofuscin granules in outer retina

Distance to ELM descent (µm)	-500	-100	↕	+100	+500*
	Non-atrophic		ELM descent	Atrophic	
**RPE phenotypes, % of locations**					
**Non-uniform**	28.6	18.8		0.0	NA
**Very non-uniform**	57.1	43.8		0.0	NA
**Sloughed †**	0.0	25.0		0.0	NA
**Shedding**	14.3	6.3		0.0	NA
**Bilaminar**	0.0	0.0		0.0	NA
**Dissociated**	0.0	0.0		56.3	NA
**Intraretinal**	0.0	0.0		0.0	NA
**Atrophy with BLamD**	0.0	0.0		43.8	NA
**Atrophy without BLamD**	0.0	0.0		0.0	NA
**M/L granules, % of locations**					
**No M/L granules**	100.0	87.5		56.3	NA
**M/L granules in OPL/HFL and/or ONL ‡**	0.0	12.5		43.8	NA
**Absent OPL/HFL/ONL**	0.00	0.00		0.00	NA

Measured at 46 locations.

ELM, external limiting membrane; M/L, melanosome/lipofuscin; OPL, outer plexiform layer; HFL, Henle fiber layer; ONL, outer nuclear layer; RPE, retinal pigment epithelium.

*The measurements at +500 are not available because the absence of a continuous RPE layer is less than 1000 µm.

†The distribution of sloughed RPE in the comparison group (13 GA eyes) is 4.7% at -500 µm and 10% at -100 µm.

‡Data from our GA database: 22.9% at -500 µm, 37.1% at -100 µm, and 72.4% at +100 µm.
