# Supplementary material for: CLINICOPATHOLOGIC CORRELATION OF GEOGRAPHIC ATROPHY SECONDARY TO AGE-RELATED MACULAR DEGENERATION
Source: Retina. 2019 Feb 6;39(4):802–16. doi: 10.1097/IAE.0000000000002461 (PMC6445604; doi:10.1097/IAE.0000000000002461)
Supplement: SUPPLEMENTARY MATERIAL [file retina-39-802-s007.pdf]

Supplementary Table 3. Stages of choriocapillaris degeneration

Distance to ELM descent, µm / ChC degeneration (% of locations)	-500	-100	↕	+100	+500
	Non-atrophic		ELM descent	Atrophic	
**Unremarkable***	50.0	68.8		62.5	NA
**Retracted**	14.3	12.5		12.5	NA
**Ghost with cells**	7.1	12.5		6.3	NA
**Ghost**	28.6	6.3		18.8	NA
**De-pillared†**	0.0	0.0		0.0	NA

Measured at 46 locations.

ELM, external limiting membrane; ChC, choriocapillaris; NA, not available.

*Data from our GA database: 58.3% at -500 µm, 35.5% at -100, 23.9% at +100 µm.

†Data from our GA database: 0.0% at -500 µm, 3.2% at -100, 15.2% at +100 µm.
