# Supplementary material for: CLINICOPATHOLOGIC CORRELATION OF GEOGRAPHIC ATROPHY SECONDARY TO AGE-RELATED MACULAR DEGENERATION
Source: Retina. 2019 Feb 6;39(4):802–16. doi: 10.1097/IAE.0000000000002461 (PMC6445604; doi:10.1097/IAE.0000000000002461)
Supplement: SUPPLEMENTARY MATERIAL [file retina-39-802-s008.pdf]

Supplementary Table 4. Outer retinal cellular and laminar phenotypes and thicknesses

**Distance to ELM descent, µm**	**-500**	**-100**	**↕**	**+100**	**+500***	***p***
	**Non-atrophic**		**ELM descent**		**Atrophic**	
**Ectopic photoreceptor nuclei, % of locations†**						
No ectopic nuclei	21.4	25.0		43.8	NA	-
Ectopic nuclei in IS	64.3	37.5		0.0	NA	-
Ectopic nuclei in OPL/HFL	57.1	75.0		56.3	NA	-
Absent OPL/HFL/ONL or dyslamination	0.0	0.0		0.0	NA	-
**ONL phenotypes, % of locations**						
Unremarkable ONL	7.1	0.0		0.0	NA	-
Thinned ONL	64.3	6.3		0.0	NA	-
Retracted nuclei & mitochondria	28.6	93.8		37.5	NA	-
Dyslaminated HFL/ONL	0.0	0.0		0.0	NA	-
ORT/ island	0.0	0.0		0.0	NA	-
Absence of continuous ONL	0.0	0.0		62.5	NA	-
**ONL/ISmy thicknesses, µm**						
ONL thickness	20.1 ± 3.7	20.1 ± 4.8		0.0 ± 0.0	NA	**<0.0001**
ONL rows (Median, IQR)	3, 0	2, 1		0, 0	NA	**-**
ISmy thickness	5.0 ± 2.0	2.4 ± 2.1		0.0 ± 0.0	NA	**<0.0001**

Measured at 46 locations.

OPL, outer plexiform layer; HFL, Henle fiber layer; ONL, outer nuclear layer; ELM, external limiting membrane; IS, inner segment; my, myoid; ORT, outer retinal tubulation; RPE, retinal pigment epithelium; IQR, interquartile range; NA, not available.

Most HFL were affected by post-mortem artifacts, so they are not included in this table.

P value in bold < 0.05.

*The measurements at +500 are not available because the absence of a continuous RPE layer is less than 1000 µm.

†These categories are not mutually exclusive, which means ectopic nuclei can locate in OPL/HFL and IS at the same time.
